# The latent net effectiveness of institutional complexes: a heuristic model

**DOI:** 10.1098/rsta.2023.0161

**Published:** 2024-04-15

**Authors:** Ashok Vardhan Adipudi, Rakhyun E. Kim

**Affiliations:** Copernicus Institute of Sustainable Development, Utrecht University, Princetonlaan 8a, 3584 CB Utrecht, The Netherlands

**Keywords:** global governance, international institutions, regime complexes, governance architectures, institutional complexity, networks

## Abstract

International institutions strive to achieve their own objectives while operating within a complex network of interdependencies. These interdependencies create an extensive web of relationships that serve as potential pathways for broader institutional impacts. The actions taken by individual institutions, their mutual influences, and the pattern of connectivity collectively shape the overall performance of institutional complexes. Understanding the performance of these complexes is crucial, yet we currently lack a methodology to assess it. To address this gap, we have developed a conceptual framework that integrates three distinct areas of study on three different scales: institutional effectiveness, institutional interlinkages, and institutional networks. This framework enables us to consider what we call the latent net effectiveness, or collective problem-solving potential, of a group of interconnected institutions. To put this framework into practice, we have devised a heuristic model, drawing from the extensive literature on international environmental institutions. As an illustrative example, we have applied this model to a network of 378 multilateral environmental agreements with 810 known issue linkages. Our work underscores the relevance of considering the system-level properties of institutional complexes and emphasizes the need for timely network-based governance and policy interventions to enhance the overall effectiveness of institutional complexes.

This article is part of the theme issue ‘A complexity science approach to law and governance'.

## 1. Introduction

International institutions form complex systems [1–3]. These systems consist of autonomous institutional elements that interact with each other, giving rise to emergent behaviour. Consider, for example, the hundreds of multilateral environmental agreements and the myriad organizations they establish [4]. Each of these international institutions pursues its own objectives and exhibits varying degrees of effectiveness under different circumstances [5]. These building blocks in turn form relationships with one another [6], and the nature of these linkages and interactions can impact their effectiveness [7–9]. When viewed as a whole, these interdependencies reveal the existence of massive institutional structures in global governance [10,11]. The configuration of these structures, whether fragmented, polycentric, or complex, is a key variable with significant implications for the overall functioning of the system of international institutions [12]. In the context of the increasing (tele)coupling of the problems that international institutions seek to address, it is imperative to pay more attention to how institutional effects aggregate at the system level [13].

In this article, we introduce the concept of *latent net effectiveness* and propose a heuristic model for its estimation, specifically in the context of a network of international institutions such as regime complexes [14,15] or governance architectures [13]. Latent net effectiveness refers to the collective problem-solving potential that could theoretically be achieved if a group of international institutions operated at their full capacity, while being subjected to endogenous and exogenous constraints. Our objective is to investigate whether international institutions possess the capability to address interconnected problems when fully implemented by their member states, or if there are inherent structural limitations. Previous discussions have raised questions regarding net effectiveness, suggesting that the whole may be less than the sum of its parts when it is excessively fragmented [16–18]. The concept of net environmental benefits has also hinted at these concerns [19]. However, previous assessments have primarily relied on qualitative approaches and anecdotal evidence [20]. Here, we aim to offer a conceptual framework and a heuristic model for evaluating the latent net effectiveness of institutional complexes in quantitative terms.

In our conceptualization, the collective problem-solving potential of an institutional system is influenced by three key variables: the latent capacity of individual institutions to produce intended effects, the nature and extent of relationships between any two institutions, and the overall structural connectivity among constituent institutions. These variables correspond to the three levels of analysis in global governance research—nodes, links and networks—and the bodies of literature around them [12]. The first body of literature focuses on individual institutions, exploring the conditions under which international institutions are effective and able to achieve their intended effects [21–27], including institutional design features [28–32]. The second body of literature examines the interlinkages and interactions between institutions in dyads, exploring the structure and dynamics of relationships and their implications for institutional effectiveness [7,8,33–38]. The third body of literature focuses on larger institutional structures, often referred to as networks, complexes or architectures, and explores how certain patterns of interconnectedness are associated with system-level behaviour or outcomes, such as flexibility and adaptability [15,39–44]. In a novel approach, we bring these distinct strands of research together for a more holistic understanding that allows estimation of the latent net effectiveness of large institutional structures.

To operationalize this framework, we build a heuristic model specifically designed for the system of multilateral environmental agreements. The model is built on previous attempts to quantify institutional effectiveness and interactions [29,45–49] as well as extensive research conducted over a span of three decades on international environmental institutions [25]. The first variable, institutional capacity, is modelled as a function of resources and institutional design features [31,50], including mechanisms for dispute resolution and compliance [5,31,45,51,52]. The second variable, interaction effects, is determined by institutional interlinkages, which are quantified using measures of membership composition and overlap [8,53,54]. The influence of these two variables is moderated by the third variable, topology, which refers to the existing pattern of issue linkages among the constituent institutions [3,55]. In our analysis, we employed citations between international institutions as proxies for issue linkages [10]. Considering the various methods available in complexity science to account for network structure [56,57], we opted for network traversals. This approach effectively incorporates the structural dimension of the network into the quantified values of institutional capacity and interaction effects [58].

By applying the heuristic model to the network of 378 multilateral environmental agreements, we demonstrate its potential for theorizing the complexity and emergent properties of global governance systems. One notable finding, for example, is the nonlinear relationship between latent net effectiveness and institutional capacity. Specific interactions are also identified as critical points for intervention to improve overall performance. Our model thus allows us to go beyond simply characterizing an institutional system as structurally fragmented or polycentric, enabling us to draw inferences about how it functions as a whole. This understanding forms the basis for devising strategies to improve the system through ‘managing' [59], ‘harnessing' [60] and ‘designing' [61] institutional complexity, by intervening at various leverage points in the system [62,63]. However, it is important to note that what we present here is a work in progress that needs to be further developed through concerted efforts. The operationalization of key variables is largely constrained by existing knowledge and available data. The assumptions we make need scrutiny, and the model should be adapted and expanded alongside the growing body of literature.

Following this introduction, we present our conceptual framework for assessing latent net effectiveness, elucidating our methodology for aggregating institutional and interaction effects within a given topology. We then draw on the existing literature to construct a heuristic model. This process involves operationalizing the variables and defining their relationships in the form of mathematical equations. Using this model, we analyse a network of 378 multilateral environmental agreements and derive several novel insights. We critically reflect on the limitations and opportunities inherent in our approach, culminating in an exploration of key challenges and potential avenues for future research.

## 2. Conceptual framework

Key concepts and variables are defined, and an overview of their relationships is provided in a conceptual framework.

### (a) Latent net effectiveness

We define latent net effectiveness as the collective problem-solving potential of a group of institutions. The effectiveness of individual institutions is ‘a function of the extent to which institutions contribute to solving or mitigating the problems that lead to their creation' [64]. However, these institutional effects are inherently systemic and can cascade, either amplifying or dampening the overall outcome [9,65]. The network of connections determines the flow of effects that can continue through third and fourth institutions, which is also largely determined by the nature of the dynamics within them. Net effectiveness is the aggregate of the relative effectiveness of all interacting institutions in a governance system. It captures the emergent property that results from the cumulative effects of institutions by taking into account the ways in which institutions interact with each other. Latent net effectiveness is a similar concept, but it represents the maximum performance that the group of institutions as a whole can hypothetically reach if each institution is implemented to its full potential in a given topology. Here, net effectiveness is latent because it is a potential value that is not constrained by the actual level of implementation, but by institutional design or structural configurations.

Understanding net effectiveness is crucial because it enables us to comprehend the dynamics of interconnected institutions within complex problem-solving scenarios. These institutions collaborate to address interconnected challenges, such as globally networked environmental risks [66], interacting planetary boundaries [67], shifting environmental problems [68], and the Sustainable Development Goals [69,70]. Optimizing individual institutions in isolation may fail to make a significant difference to their collective performance, or may even undermine it. However, the effectiveness of these institutional complexes in tackling the interconnected problems remains unclear. While some argue that polycentric complexes can be effective under certain conditions [71,72], there is limited understanding of the configurations that allow for maximum synergy, surpassing the cumulative effects of individual institutions [73]. Consider, for example, the coordination required among dozens of regional fisheries management organizations [74]. Is the net effectiveness of the group greater than the sum of the effectiveness of the individual institutions? This is an important governance question for which there is no clear answer.

The concept of *latent net effectiveness* has significant analytical value. By understanding what is possible assuming full implementation, it allows us to discern the impact of design and structural constraints on net effectiveness. Measures could then be devised to improve those design and structural features. These encompass the design of individual institutions, the extent of institutional overlap or separation, and the complex pattern of institutional interlinkages. Estimating latent net effectiveness would allow us to address the following questions [75,76]. Will the myriad problems addressed by multilateral environmental agreements be sufficiently mitigated and the integrity of Earth's life-support systems restored or maintained if the agreements are fully implemented? Or are there underlying design flaws that also need fixing?

### (b) Institutional capacity, interlinkages and topology

**Figure 1.**
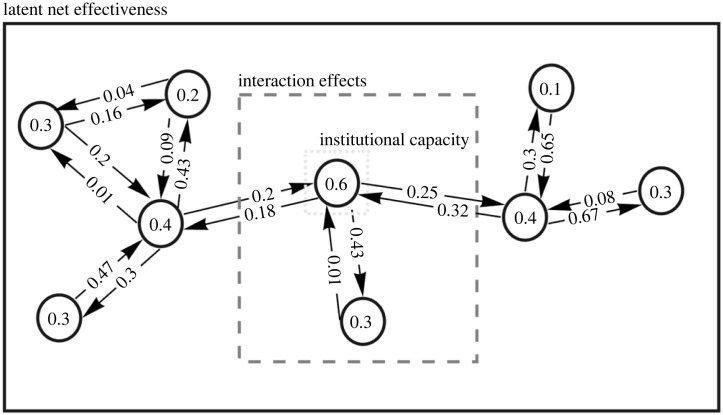
A schematic representation of the conceptual framework, consisting of individual institutions with capacity, which interact along issue linkages, but have institutional interlinkages that are fragmentated to varying degrees. Latent net effectiveness is a function of institutional capacity at the node level, institutional interlinkages at the edge level, and topology at the network level.

We conceptualize latent net effectiveness as a function of institutional capacity, institutional interaction, and the pattern of interactions (figure 1). Institutional capacity refers to the measured extent of an institution's problem-solving capacity to produce intended effects, which is, in turn, shaped by its institutional design characteristics. The capacity of an institution is also influenced by its interactions with other institutions, resulting in interaction effects. Given the interconnected nature of institutions, these interaction effects propagate and spread across multiple institutions within the network, producing a cascading effect.

At the individual institutional level of the network, institutions produce effects within the confines of their resources and design. The presence of design elements such as compliance or dispute resolution within an institution can significantly influence its problem-solving capabilities. The impact of an institution can be approximated by evaluating such latent capacity in accomplishing its objectives. For example, the impact of regional fisheries management organizations in managing fish stocks can vary depending on their capacity. While some of them may demonstrate highly positive effects, others may have less pronounced effects. The overall impact can theoretically be quantified and represented by a single value, such as on a scale ranging from 0 to 1.

At the dyadic level, institutions interact with one another and affect their institutional capacity to achieve their objectives [7,77–79]. The nature and magnitude of such interaction effects would depend on the institutional interlinkages between the two institutions concerned. For example, the degree of fragmentation in terms of shared membership is widely accepted as a key determinant [80], along with several other structural aspects such as information asymmetry [54,81]. Like institutional capacity, interaction effects can also range from 0 to 1, representing the extent to which an institution is influenced by its interactions with another institution.

At the network level, institutional interactions give rise to a network structure, where emergent properties manifest as the collective outcomes of all the interaction effects on institutional capacity. Consider, for example, a network of three institutions, which is the simplest form of a regime complex [41]. The overall performance of a network of three institutions cannot be estimated simply by adding up institutional capacities without considering how the effects may flow and cascade, or by taking a dyadic approach that does not consider the interdependencies between interactions (dyads) [82]. One needs to capture ‘the qualitative change that occurs when we move from two to three distinct entities' [41]. Topology holds significance because two groups with the same membership composition perform differently depending on the patterns of relationships between members [83].

The concepts underlying the framework and the variables involved can be summarized as follows. Institutions of different design exhibit varying capacities to achieve intended effects. The nature of the relationships between institutions gives rise to specific interactions that influence these capacities and, consequently, their latent effectiveness. This process of effect creation and adjustment takes place within the network topology, leading to the emergence of overall effects. The microcosm of the network, represented by the institutional capacity of each node, is then projected onto the macrocosm of the network, resulting in the collective impact measured in terms of latent net effectiveness.

## 3. Heuristic model

We present a heuristic model specifically designed for estimating the latent net effectiveness of international environmental treaty regimes.

### (a) Institutional capacity

Institutional capacity is defined as the latent effectiveness of an institution that can be realized when fully implemented and complied with. The literature on the effectiveness of international environmental institutions highlights certain design characteristics that are considered essential [84–86]. These features, which are treated as independent variables in the analysis of institutional effectiveness, include membership, resources or budget allocation, the existence of treaty bodies such as a secretariat and a scientific body, the presence of mechanisms such as information sharing, enforcement, compliance, and dispute settlement, as well as the capacity to make decisions and adapt through a decision-making body like a conference of the parties (COP) (table 1).

**Table 1.** Key variables used to operationalize the capacity of international institutions to produce intended effects, how they are approximated, and how they relate to institutional capacity.

variables	operationalization	relationship with institutional effects
membership (*M*)	the normalized cumulative GDP (value ranging between 0 and 1) of the member states in 2021 that have ratified the agreement. E.g. the United States is not a party to the CBD, hence *M* is 0.714	the more member states and the more resources an international institution has, the greater its capacity to produce intended effects
budget (*B*)	the normalized budget (value ranging between 0 and 1) of the institution. E.g. the UNFCCC has a budget of USD31 470 564, hence *B* is 0.000174	
scientific body (*m*)	binary variable. E.g. the UNFCCC established the Subsidiary Body for Scientific and Technological Advice, hence *m* is 1	the presence of institutional arrangements such as a secretariat, scientific body, information exchange mechanism, and enforcement mechanism increases the capacity of an international institution to produce intended effects
secretariat (*j*)	binary variable. E.g. the Basel Convention has a secretariat, hence *j* is 1	
information exchange mechanism (*p*)	binary variable. For instance, the Cartagena Protocol established the Biosafety Clearing-House, hence *p* is 1	
compliance or enforcement mechanism (*q*)	binary variable. For instance, UNCLOS with a dispute settlement mechanism has *q* of 1	
flexibility (*f*)	categorical variable with 0 (agreements with no COPs or like bodies), 1 (agreements with COPs but without provision for amendment), and 1.5 (agreements with COPs including provision for amendment) as possible values. E.g. the Basel Convention has a COP and has a provision for amendment, hence *f* is 1.5	the ability of international institutions to adapt leads to increased institutional capacity to produce intended effects

Membership in an international agreement and sufficient budgetary resources play a significant role in influencing institutional effects. The availability of resources enables an institution to effectively implement its objectives through projects or cooperative efforts [87]. As these resources typically originate from contributions made by member states, we assume that the significance of a particular member state's presence is directly proportional to its GDP. Thus, this variable is represented as a weighted sum of the GDPs of the member states belonging to a given treaty.

The presence of treaty bodies and mechanisms is indicative of a higher institutional impact. Secretariats and scientific bodies operate autonomously and play a significant role in coordinating stakeholders and fostering synergies [88–91]. Moreover, the existence of mechanisms for compliance, enforcement, dispute resolution, and other related functions is a critical aspect of effective institutional design to enhance effectiveness [26]. They tend to promote the implementation of and compliance with key obligations by member states [31,92–95].

The ability of an institution to navigate unforeseen and evolving circumstances is important for enhancing institutional effectiveness. In this context, flexibility refers to the capacity to change and adapt, allowing institutions the necessary space to transform themselves and potentially adjust their objectives to remain relevant [84,96–98]. This adaptability can be facilitated through amendments and decisions by treaty bodies, such as a COP. Flexibility also ensures that institutions can overcome political impasses and continue to function effectively [84,98].

Based on this equation and the relationships identified in table 1, we arrive at equation (3.1):

3.1 Institutional capacity ∝(M+B)×(1+m+j+p+q+f).

Equation (3.1) represents the variables involved in institutional design and their interrelationships. The two clusters of variables, namely (*M* + *B*) and (1 + *m* + *j* + *p* + *q* + *f*), capture key aspects of institutional design, including member states and resources, subsidiary bodies and institutional flexibility. These variables converge to produce a quantified measure of institutional capacity, encompassing elements that replicate the diverse facets of institutional design and their implications for institutional effectiveness. This structure of equation (3.1) allows for the consideration of factors such as the balance between adaptable and coordinated functioning and certainty, which influence the varying levels of effective outcomes achieved through agreements. Both aspects are reflected in the combination of independent variables: flexibility (*f*) and subsidiary bodies excluding enforcement mechanisms (*m*, *j* and *p*) on the one hand, and enforcement mechanisms (*q*) on the other.

### (b) Interaction effects

The institutional capacity to achieve its objectives can be undermined by the level of institutional cooperation or fragmentation. For example, the ability to fully protect biodiversity can only be realized if institutions addressing climate change cooperate [99]. In a dyadic institutional interaction, the extent of this cooperation is structurally constrained by the degree of institutional fragmentation in the interlinkage between institutions that share overlapping issues. We measure institutional interlinkages in terms of fragmentation, which is determined by two variables: the extent of membership overlap and the homogeneity of members' interests (table 2). This measures how well two institutions cooperate on the basis of their membership characteristics. Interaction effects thus affect their relative effectiveness. Any interaction effects are also proportional to the institutional capacity of the source institution, which is the outcome of equation (3.1).

**Table 2.** Independent variables for institutional interlinkages, their operationalization and relationship with interaction effects.

variables	operationalization	relationship with interaction effects
degree of membership overlap (MO)	an overlap is given a value between 0 and 1, where 0 indicates no overlap in membership and 1 indicates complete membership overlap. The edges are directional, so is the effect. The overlap calculation uses GDP-weighted overlap in this directional sense. E.g. all the parties to the 1945 supplementary protocol to the Whaling Convention are parties to the convention but not the other way around. Therefore, membership overlap is 1 and 0.89, respectively	full membership overlap maximizes the possibility of a cooperative influence from one institution to another. The extent of non-overlap is proportional to the possibility of institutional undermining
homogeneity of member interests (HMI)	the homogeneity of members' interests yields values between 0 and 1, where 0 indicates diversity among members and 1 indicates homogeneity of members. Normalized standard deviation among the member states’ GDP per capita yields HMI. E.g. the agreement for the establishment of a Commission for Controlling the Desert Locust in Southwest Asia has a very high HMI of 0.98	internal diversity limits the potential collective approaches to engagement with another institution, resulting in a tendency to avoid engagement altogether. Conversely, homogeneity tends to encourage a wider range of possibilities for interaction

The degree of membership overlap (MO) serves as a significant indicator of institutional fragmentation, determining the potential for one institution to undermine another [42]. When a member state belongs to only one institution but not both, it can hinder the possibility of cooperation between the two institutions [38]. The impact of this fragmentation is asymmetric and varies depending on the direction of influence. For instance, all parties to the Convention on Biological Diversity (CBD) are also parties to the United Nations Framework Convention on Climate Change (UNFCCC), resulting in complete membership overlap and no fragmentation from the perspective of the CBD. Consequently, the CBD does not undermine the UNFCCC. However, from the perspective of the UNFCCC, there is no complete membership overlap because the United States is not a party to the CBD, resulting in a partial disconnect that undermines the institutional capacity or latent effectiveness of the CBD. The impact is determined by the extent of non-overlap and the political power of obstructing states. While all states have equal legal standing, the refusal of the Holy See to consider biodiversity issues at a climate COP would not carry the same impact as the United States doing the same. The differential impact can be operationalized by considering the GDP of each state.

The homogeneity of members' interests plays a significant role in institutional interactions [79,84]. When an institution is internally divided, it is more likely to undermine or fail to cooperate with another institution. For instance, if a treaty includes members from both the Global North and the Global South, their divergent views on how to collectively engage with another institution addressing a different but related issue may lead to non-engagement, deepening fragmentation. On the other hand, homogeneity of member interests tends to have the opposite effect. When member interests are aligned, institutions are more likely to cooperate and engage effectively. To measure the homogeneity of member interests, we operationalize it as the absence of discrepancies in GDP per capita among member states, which can be measured by the standard deviation.

Based on this equation and the relationships identified in table 2, we arrive at equation (3.2):

3.2 Interaction effects∝Institutional capacity×(MO+HMI).

It is important to note that these variables are institutional in nature and do not directly indicate the presence of issue overlap. We do not assume that interaction occurs simply because there is overlap in membership. The presence of issue overlap depends on exogenous factors, including interdependencies between different issue areas, such as climate change and biodiversity conservation. When there is an issue linkage between two international institutions, the equation above can be used to assess the effects of this interlinkage on institutional capacity. Furthermore, the choice and operationalization of variables can be adapted to the application at hand and to the data available.

### (c) Topology

We assume that international institutions interact when the issues they address are linked. Identifying issue linkages between international institutions can be done in many ways, in particular by reviewing the literature on the issues themselves [12]. Here, we use citations as a proxy for issue linkages. In other words, we consider issue linkages to exist between two international institutions and these institutions to interact if one institution explicitly mentions the other by name in its founding document [10,11]. The effects of these interactions are not determined by citations; equation (3.2) above aims to estimate these effects.

After mapping all issue linkages, we use network tracing to aggregate the influence of interaction effects on institutional capacity in a given topology. This approach uses the literal structure of the network by traversing from one node to another, considering a set of edges, and calculating the collective weights of institutional and interaction effects [8,58]. Network traversal allows us to capture not only first-order interactions but also higher-order interactions, thereby accounting for ‘the systemic impact of one target beyond its closest neighbours' [100] and bridging ‘the micro-macro gap’ [101]. This process accounts for qualitative changes between nodes within the network topology by calculating the multiplied collective weight during each traversal [102].

To aggregate the values obtained from network traversals and estimate latent net effectiveness, we employ an iterative approach to implement random walks originating from each node within the network [103,104]. This estimation is achieved by averaging the outputs obtained from traversing the network, where the value of the traversal in each iteration represents the average value of randomly selected traversals from each network node or international institution. This iterative process of conducting random walks from each node generates multiple sub-structures within the network, each of which would otherwise be unique and difficult to isolate. By continually traversing the network and correcting the parameters based on their values at each node relative to the effects, this method effectively accounts for the intricate structural interconnections inherent in the complex network.

Consequently, the macro-level aspect of our framework becomes operationalized and functions at the intersection of the body of research concerning qualitative aspects of institutional and interaction effects within the multilateral environmental agreements network outlined earlier. It is important to note, however, that the use of the average of weighted network traversals as a measure of latent net effectiveness may not always be the optimal choice. For instance, when investigating lower-order effects of interactions within the complex network, it may be more advantageous to consider the maximum values obtained from random walks. Therefore, the choice of measurement may necessitate adaptation to align with the specific research question at hand.

## 4. Application

**Figure 2.**
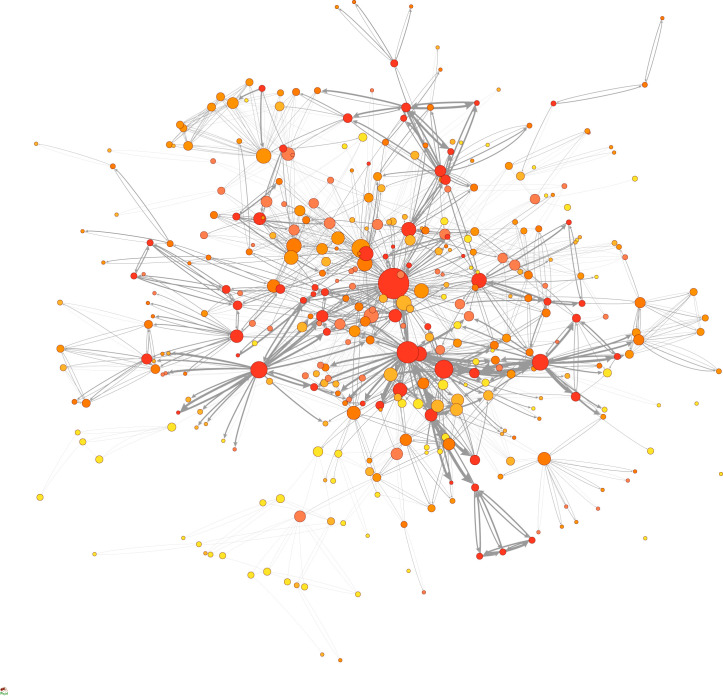
The network of 378 multilateral environmental agreements with 810 issue overlaps. The size of the nodes represents in-degree. The colours of the nodes from lighter to darker shades and the width of the edges from thinner to thicker represent increasing values of institutional capacity and interaction effects. (Online version in colour.)

For illustrative purposes, we applied our heuristic model to the network of multilateral environmental agreements and estimated its latent net effectiveness (figure 2). We used the International Environmental Agreements Database as the primary source of data on the agreements [4]. We first selected the agreements with membership data, and coded them (see electronic supplementary material). Citation data were obtained from Kim [10] as updated in Kim & Morin [11]. We zoomed in on the largest connected component of the network as the object of analysis. This is a network of 378 multilateral environmental agreements adopted between 1919 and 2014. Overall, the network has a complex structure with communities, relatively low average path length, high clustering coefficient, and skewed degree distribution.

The average institutional capacity of these agreements is 0.502 with a median of 0.204. Among the most capable agreement is the UNFCCC and among the least include the Agreement Establishing the Caribbean Community Climate Change Centre. These agreements are connected through 810 issue linkages or 1620 directed edges. Of the 1620 directed edges, 202 have no membership overlap at all (12% of directed edges, and 53% of nodes), while 230 have complete membership overlap (14% of directed edges, and 67% of nodes).

The estimated value of the latent net effectiveness of this network of multilateral environmental agreements is 0.000156. To obtain this value, we ran 10 000 iterations of random walks on the network from each node in the network. The values of institutional and interaction effects are relative to other values at their respective structural levels of the network (and therefore constantly changing) and are not absolute. To make sense of this value, we compared it with the values we get when we run calculations on a few hypothetical scenarios. These scenarios help us understand the direction of change vis-à-vis a given variable. They include intervening at a few specific central nodes, increasing the capacity of all nodes, and fragmenting and defragmenting the system.

**Figure 3.**
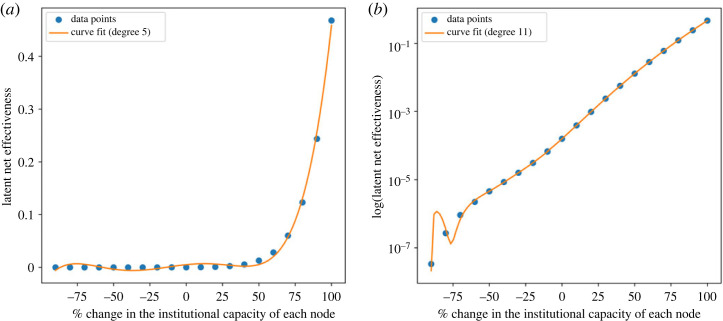
(*a*) Latent net effectiveness versus % increase in institutional capacity of each node. Data points fit the fifth-degree polynomial curve. (*b*) Log of latent net effectiveness versus % increase in institutional capacity of each node. Data points fit the eleventh-degree polynomial curve. (Online version in colour.)

**Figure 4.**
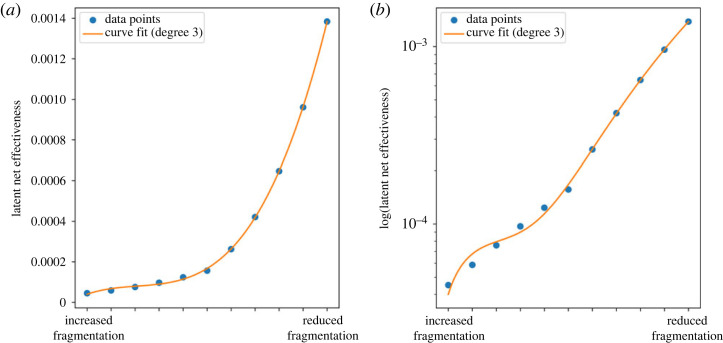
(*a*) Latent net effectiveness versus fragmentation level across institutional pairs. Data points fit the third-degree polynomial curve. (*b*) Log of latent net effectiveness versus fragmentation level across institutional pairs. Data points fit the third-degree polynomial curve as well. (Online version in colour.)

We found an exponential relationship between institutional capacity and latent net effectiveness (figure 3). This nonlinear increase is noticeable from the knee point around 50% increase in institutional capacity. In addition, a change in the level of fragmentation also produces a nonlinear response in latent net effectiveness (figure 4). Latent net effectiveness is currently at its minimum relative to the degree of fragmentation, and any reduction in fragmentation will result in a significant increase in the overall capacity of the treaty system to produce intended effects.

We also observe that latent net effectiveness diminishes as institutional capacity decreases (see the logarithmic plot in figure 3*b*). This decline is significantly more pronounced than the increase associated with an augmentation in institutional capacity. This places latent net effectiveness at a key juncture, where interventions aimed at altering the system can yield either highly favourable or unfavourable outcomes for the system. It is evident that such a transformation must encompass the entire system. Isolated adjustments in institutional capacity or fragmentation alone cannot surmount the threshold required for inducing radical change. But a 50% change in institutional capacity throughout the network's nodes can yield an astonishing 82-fold shift in latent net effectiveness. A gradual reduction in fragmentation or an escalation in institutional capacity across the board can generate a positive impact, eventually leading to a substantial transformation in the state of the system as the interventions progress. Therefore, extant structural elements can be leveraged to enhance the network's overall problem-solving capacity.

Regarding policy implications, the simulations underscore the importance of prioritizing the reduction of fragmentation over exclusively focusing on enhancing institutional capacity. The simulations reveal that even a 50% boost in institutional capacity may not result in substantial improvements in latent net effectiveness. Conversely, any reduction in the degree of fragmentation would yield immediate and progressive enhancements in latent net effectiveness. Furthermore, targeted interventions aimed at pivotal nodes within the network can have a substantial impact on latent net effectiveness. For example, the United States ratifying both the CBD and the United Nations Convention on the Law of the Sea leads to a notable 6% upswing in latent net effectiveness.

## 5. Conclusion

This study responds to the call made by Gehring and Oberthür over 15 years ago to ‘grasp complex interaction situations and start exploring their emergent properties' [8]. Understanding the conditions under which emergent properties, such as latent net effectiveness, vary is imperative. The issues that international institutions seek to address are increasingly interconnected [105,106], and the institutions themselves are dynamic and interacting, making the institutional landscape ever more complex. Efforts have been made to unravel this institutional complexity by decomposing it into bilateral cases [107] or by mapping the network topology [11,106]. Building on this, we made an attempt at piecing together these fragmented parts to gain a comprehensive understanding of the whole system.

We started at the level of international institutions, where different institutional designs lead to varying capacities to produce intended effects. These institutions then interact with others, but depending on the structural constraints of their interlinkages, some institutions may undermine the possibility of cooperation more than others. Such undermining has an impact on the capacity of individual institutions. At the network level, the aggregation of institutional and interaction effects determines its latent net effectiveness. Yet it is not the simple sum of these effects, as we need to take into account the patterns of relationships or topology [108,109]. In our model, the potential effects of individual institutions and their interactions are first approximated and then aggregated using random walks along network paths.

Our research indicates that an understanding of network-level properties, such as latent net effectiveness, can be useful for policymakers. In particular, these properties would be instrumental when coordinating international institutions that navigate complex trade-offs while pursuing their own policy objectives [61,110,111]. In such a context, optimizing individual institutions in isolation may not lead to the most optimal outcome for the system as a whole. Here, latent net effectiveness serves as a valuable reference point or system-level indicator. The application of our heuristic model to the network of multilateral environmental agreements has demonstrated this. Understanding the factors that contribute to or hinder latent net effectiveness will aid in tackling what is essentially the challenge of multi-objective optimization in global governance. Measuring latent net effectiveness is, therefore, a critical step towards harnessing institutional complexity [60] and addressing globally networked risks [112–114].

However, the efficacy of such parameters in guiding policy and decision making depends on our ability to refine the modelling of institutional functioning [115]. While the proposed framework has strengths, particularly in its comprehensive approach to network topology and its adaptability in formulating parameters of interest, it remains reliant on the underlying institutional theories for qualitative accuracy. The selection and operationalization of variables inevitably involve a degree of subjectivity, underscoring the need to tailor them to the specific application and available data. Furthermore, the variables included in the model can never be exhaustive, as there will always be other factors that are not considered. One of these factors, for instance, is the problem structure, which refers to the set of variables that define the nature and characteristics of the problems under consideration. It serves as an exogenous factor alongside the endogenous factors within the system itself [52,116]. However, the relationship between institutional design and problem structure remains unclear in the existing literature [50,52]. This has prevented us from including a quantitative variable for problem structure in our heuristic model. Another limitation stems from the lack of specific weights assigned to the variables in our model. The relative importance of variables would vary across different domains of global governance [26], and assigning weights to variables would furnish a more nuanced understanding of specific applications. Furthermore, latent net effectiveness may also hinge on additional system-level variables such as resilience or redundancy, which were not included in the model or analysis. The limitations discussed here point to potential avenues for future research [117].

The profound challenge of the Anthropocene revolves around enhancing the net effectiveness of international institutions to address intricately interlinked global issues. We extend an earnest invitation to shift our perspective beyond the conventional emphasis on isolated components and one-on-one interactions, and instead, adopt the governance paradigm as a matter of multi-objective optimization. In this framework, the paramount query becomes whether the entirety of Earth system governance surpasses or lags behind the cumulative influence of its individual elements, and how we can intervene to enhance their collective performance.

## Data Availability

The data are provided in electronic supplementary material [118].
